# Supplementary material for: Comparative effectiveness of glucagon-like peptide-1 receptor agonists on body composition and anthropometric indices: A protocol for a systematic review and network meta-analysis of randomized controlled trials
Source: PLoS One. 2024 Feb 26;19(2):e0297488. doi: 10.1371/journal.pone.0297488 (PMC10896528; doi:10.1371/journal.pone.0297488)
Supplement: S2 Table — (PDF) [file pone.0297488.s002.pdf]

S2 Table Systematic Review Search Strategy

**Ovid MEDLINE(R) ALL 1946 to September 02, 2023**		
**Search**	**Query**	**Items Found**
#1	exp obesity/	262047
#2	exp overweight/	273447
#3	exp body weight changes/	83972
#4	exp weight reduction programs/	3074
#5	exp weight loss/	49517
#6	exp body weight/	533406
#7	exp body composition/	63972
#8	exp body fat distribution/	17943
#9	exp body mass index/	149482
#10	exp waist-hip ratio/	4583
#11	body weight loss.mp.	6914
#12	((body mass index or BMI or body weight) adj3 over).tw.	4156
#13	(weight adj2 (loss or lost or losing or reduc$ or gain$ or increas$ or program*)).tw.	230965
#14	(obes$ or overweight or over-weight or weight loss or weight reduction or body mass index or high BMI or BMI above).ti,ab,kf.	631437
#15	or/1-14	998889
#16	exp diabetes mellitus type 2/	172439
#17	diabetes mellitus.mp.	533780
#18	NIDDM.tw.	6960
#19	exp diabetic nephropathy/	29614
#20	(diabetic nephropath* or diabetic kidney disease).tw.	25719
#21	((diabetes or diabetes mellitus or diabetic*) adj1 (type 2 or type II or type ii or non-insulin dependent or noninsulin dependent or adult onset or mature onset or late onset)).ti,ab,kf.	198894
#22	or/16-21	585120
#23	15 or 22	1455513
#24	exp receptors, glucagon/	2560
#25	exp glucagon like peptide 1/	11120
#26	((glucagon-like peptide or glucagon-like peptide 1 or GLP-1 or GLP1) adj1 (receptor* or stimulat* or inhibitor* or agonist* or long act*)).ti,ab,kf.	7204
#27	glucose-dependent insulinotropic polypeptide.mp.	1900
#28	(glucose-dependent insulinotropic polypeptide or GIP) adj1 (inhibitor* or agonist*).ti,ab,kf.	40
#29	(albiglutide or GSK-716155 or GSK716155).ti,ab,kf.	230
#30	(dulaglutide or LY-2189265 or LY2189265).ti,ab,kf.	699
#31	(efpeglenatide or HM-11260C or SAR-439977 or SAR439977).ti,ab,kf.	32
#32	(exenatide or exendin 4 or AC-2993 or AC2993 or LY-2148568 or LY2148568).ti,ab,kf.	4105
#33	(liraglutide or NN-2211 or NN2211).ti,ab,kf.	3863
#34	(lixisenatide or AVE-010 or AVE-0010 or AVE0010 or AVE010).ti,ab,kf.	561
#35	(semaglutide or NN-9535 or NN9535 or NN9924 or NN-9924).ti,ab,kf.	1228
#36	(taspoglutide or BIM51077 or RO-5073031 or RO5073031).ti,ab,kf.	60
#37	(tirzepatide or LY3298176 or LY-3298176 or LY 3298176).ti,ab,kf.	277
#38	or/24-37	20767

#39	23 and 38	14567
#40	exp Clinical Trials as Topic/	384161
#41	(randomi$ed controlled trial* or controlled clinical trial*).mp.	133053
#42	((random allocation) or (double-blind method) or (single-blind method) or (clinical trial)).mp.	967187
#43	((controlled clinical trial) or (randomi$ed controlled trial) or (clinical trial)).pt	563024
#44	(control* adj2 trial*).tw,kw.	369856
#45	((clinical adj trial*) or (randomly allocated) or (allocated adj2 random*) or randomi$ed or RCT$1 placebo*).tw,kw.	518989
#46	((singl* or doubl* or trebl* or tribl*) adj (blind* or mask* or dumm*)).tw,kw.	197687
#47	or/40-46	1651684
#48	39 and 47	3912
#49	limit 48 to human	3320
#50	exp Adolescent/ not (exp Adult/ and Adolescent/)	688261
#51	exp Child/ not (exp Adult/ and exp Child/)	1396245
#52	exp Infant/ not (exp Adult/ and exp Infant/)	923938
#53	or/50-52	2148226
#54	49 not 53	3277

S2 Table Systematic Review Search Strategy (Continued)

**Embase (Elsevier) 1980 to September 02, 2023**		
**Search**	**Query**	**Items Found**
#1	'obesity'	744763
#2	overweight/exp AND [embase]/lim	589149
#3	'body weight changes'/exp AND [embase]/lim	338664
#4	'weight reduction programs'/exp AND [embase]/lim	2617
#5	'weight loss'/exp AND [embase]/lim	222787
#6	'body weight'/exp AND [embase]/lim	773294
#7	'body composition'/exp AND [embase]/lim	108484
#8	'body fat distribution'/exp AND [embase]/lim	7473
#9	'body mass index'/exp AND [embase]/lim	597483
#10	'waist-hip ratio'/exp AND [embase]/lim	18025
#11	'body weight loss'/exp AND [embase]/lim	222787
#12	(('body mass index':ti,ab OR BMI:ti,ab OR 'body weight':ti,ab) AND (over:ti,ab)) AND [embase]/lim	102291
#13	((weight:ti,ab) AND (loss:ti,ab OR lost:ti,ab OR losing:ti,ab OR reduc*:ti,ab OR gain*:ti,ab OR increase*:ti,ab OR program*:ti,ab)) AND [embase]/lim	726699
#14	(obes*:ti,ab OR overweight:ti,ab OR 'over-weight':ti,ab OR 'weight loss':ti,ab OR 'weight reduction':ti,ab OR 'body mass index':ti,ab OR 'high BMI':ti,ab OR 'BMI above':ti,ab) AND [embase]/lim	810980
#15	#1 OR #2 OR #3 OR #4 OR #5 OR #6 OR #7 OR #8 OR #9 OR #10 OR #11 OR #12 OR #13 OR #14	2074846
#16	'diabetes mellitus type 2'/exp AND [embase]/lim	303118
#17	'diabetes mellitus'/exp AND [embase]/lim	1124946
#18	NIDDM/exp AND [embase]/lim	303118
#19	'diabetic nephropath*'/exp AND [embase]/lim	54268
#20	('diabetic nephropath*':ti,ab OR 'diabetic kidney disease':ti,ab) AND [embase]/lim	33993
#21	((diabetes:ti,ab OR 'diabetes mellitus':ti,ab OR diabetic*:ti,ab) AND ('type 2':ti,ab OR 'type II':ti,ab OR 'type ii':ti,ab OR 'non-insulin dependent':ti,ab OR 'noninsulin dependent':ti,ab OR 'adult onset':ti,ab OR 'mature onset':ti,ab OR 'late onset':ti,ab)) AND [embase]/lim	281946
#22	#16 OR #17 OR #18 OR #19 OR #20 OR #21	1153239
#23	#15 OR #22	2845190
#24	'receptors, glucagon'/exp AND [embase]/lim	6901
#25	'glucagon like peptide 1'/exp AND [embase]/lim	22817
#26	(('glucagon-like peptide':ti,ab OR 'glucagon-like peptide 1':ti,ab OR 'GLP-1':ti,ab OR GLP1:ti,ab) AND (receptor*:ti,ab OR stimulat*:ti,ab OR inhibitor*:ti,ab OR agonist*:ti,ab OR 'long act*':ti,ab)) AND [embase]/lim	22669
#27	'glucose-dependent insulinotropic polypeptide'/exp AND [embase]/lim	6497
#28	('glucose-dependent insulinotropic polypeptide':ti,ab OR GIP:ti,ab) AND (inhibitor*:ti,ab OR agonist*:ti,ab) AND [embase]/lim	2887
#29	(albiglutide:ti,ab OR 'GSK-716155':ti,ab OR GSK716155:ti,ab) AND [embase]/lim	389
#30	(dulaglutide:ti,ab OR 'LY-2189265':ti,ab OR LY2189265:ti,ab) AND [embase]/lim	1349
#31	(efpeglenatide:ti,ab OR 'HM-11260C':ti,ab OR 'SAR-439977':ti,ab OR SAR439977:ti,ab) AND [embase]/lim	61

#32	(exenatide:ti,ab OR ‘exendin 4’:ti,ab OR ‘AC-2993’:ti,ab OR AC2993:ti,ab OR ‘LY-2148568’:ti,ab OR LY2148568:ti,ab) AND [embase]/lim	6613
#33	(liraglutide:ti,ab OR ‘NN-2211’:ti,ab OR NN2211:ti,ab) AND [embase]/lim	6875
#34	(lixisenatide:ti,ab OR ‘AVE-010’:ti,ab OR ‘AVE-0010’:ti,ab OR AVE0010:ti,ab OR AVE010:ti,ab) AND [embase]/lim	912
#35	(semaglutide:ti,ab OR ‘NN-9535’:ti,ab OR NN9535:ti,ab OR NN9924:ti,ab OR ‘NN-9924’:ti,ab) AND [embase]/lim	2153
#36	(taspoglutide:ti,ab OR BIM51077:ti,ab OR ‘RO-5073031’:ti,ab OR RO5073031:ti,ab) AND [embase]/lim	99
#37	(tirzepatide:ti,ab OR LY3298176:ti,ab OR ‘LY-3298176’:ti,ab OR ‘LY 3298176’:ti,ab) AND [embase]/lim	458
#38	#24 OR #25 OR #26 OR #27 OR #28 OR #29 OR #30 OR #31 OR #32 OR #33 OR #34 OR #35 OR #36 OR #37	45106
#39	#23 AND #38	34685
#40	#39 AND ([adult]/lim OR [aged]/lim OR [middle aged]/lim OR [very elderly]/lim OR [young adult]/lim)	12038
#41	#40 AND ('clinical trial'/de OR 'clinical trial topic'/de OR 'comparative study'/de OR 'controlled clinical trial'/de OR 'controlled study'/de OR 'double blind procedure'/de OR 'parallel design'/de OR 'phase 2 clinical trial'/de OR 'phase 3 clinical trial'/de OR 'phase 3 clinical trial topic'/de OR 'pilot study'/de OR 'randomized controlled trial'/de OR 'randomized controlled trial topic'/de OR 'single blind procedure'/de)	9280

S2 Table Systematic Review Search Strategy (Continued)

**PubMed (From Inception to September 02, 2023)**		
**Search**	**Query**	**Items Found**
#1	abdominal obesity[MeSH Terms]	5230
#2	overweight[MeSH Terms]	272356
#3	body weight changes[MeSH Terms]	83951
#4	programs, weight reduction[MeSH Terms]	3074
#5	agents, weight loss[MeSH Terms]	9947
#6	body weight[MeSH Terms]	533273
#7	body compositions[MeSH Terms]	63949
#8	body fat distribution[MeSH Terms]	17937
#9	body mass index[MeSH Terms]	149452
#10	ratio, waist to hip[MeSH Terms]	4582
#11	("body mass index"[Title/Abstract] OR BMI[Title/Abstract] OR "body weight"[Title/Abstract]) AND (over[Title/Abstract])	61667
#12	(weight[Title/Abstract]) AND (loss[Title/Abstract] OR lost[Title/Abstract] OR losing[Title/Abstract] OR reduc*[Title/Abstract] OR gain*[Title/Abstract] OR increase*[Title/Abstract] OR program*[Title/Abstract])	605819
#13	obes*[Title/Abstract] OR overweight[Title/Abstract] OR "over-weight"[Title/Abstract] OR "weight loss"[Title/Abstract] OR "weight reduction"[Title/Abstract] OR "body mass index"[Title/Abstract] OR "high BMI"[Title/Abstract] OR "BMI above"[Title/Abstract]	631490
#14	#1 OR #2 OR #3 OR #4 OR #5 OR #6 OR #7 OR #8 OR #9 OR #10 OR #11 OR #12 OR #13	1288706
#15	adult onset diabetes mellitus[MeSH Terms]	172381
#16	"diabetes mellitus type 2"[Title/Abstract] OR "diabetes mellitus"[Title/Abstract] OR NIDDM[Title/Abstract]	262761
#17	diabetic nephropathy[MeSH Terms]	29601
#18	diabetic nephropath*[Title/Abstract] OR diabetic kidney disease[Title/Abstract]	26854
#19	(diabetes[Title/Abstract] OR "diabetes mellitus"[Title/Abstract] OR diabetic*[Title/Abstract]) AND ("type 2"[Title/Abstract] OR "type II"[Title/Abstract] OR "type ii"[Title/Abstract] OR "non-insulin dependent"[Title/Abstract] OR "noninsulin dependent"[Title/Abstract] OR "adult onset"[Title/Abstract] OR "mature onset"[Title/Abstract] OR "late onset"[Title/Abstract])	208407
#20	#15 OR #16 OR #17 OR #18 OR #19	433935
#21	#14 OR #20	1606845
#22	glucagon like peptide 1[MeSH Terms]	11113
#23	glucose dependent insulinotropic peptide[MeSH Terms]	2988
#24	"glucagon receptor"[Title/Abstract] OR "glucagon like peptide 1"[Title/Abstract] OR "glucose-dependent insulinotropic polypeptide"[Title/Abstract]	16977
#25	("glucagon-like peptide"[Title/Abstract] OR "glucagon-like peptide 1"[Title/Abstract] OR "GLP-1"[Title/Abstract] OR GLP1[Title/Abstract]) AND (receptor*[Title/Abstract] OR stimulat*[Title/Abstract] OR inhibitor*[Title/Abstract] OR agonist*[Title/Abstract] OR "long act*"[Title/Abstract])	15731

#26	("glucose-dependent insulinotropic polypeptide"[Title/Abstract] OR GIP[Title/Abstract]) AND (inhibitor*[Title/Abstract] OR agonist*[Title/Abstract])	2149
#27	albiglutide OR GSK-716155 OR GSK716155	264
#28	dulaglutide OR LY-2189265 OR LY2189265	729
#29	efpeglenatide OR HM-11260C OR SAR-439977 OR SAR439977	33
#30	exenatide OR "exendin 4" OR AC-2993 OR AC2993 Or LY-2148568 OR LY2148568	4443
#31	liraglutide OR NN-2211 OR NN2211	4127
#32	lixisenatide OR AVE-010 OR AVE-0010 OR AVE0010 OR AVE010	588
#33	semaglutide OR NN-9535 OR NN9535 OR NN9924 OR NN-9924	1301
#34	taspoglutide OR BIM51077 OR RO-5073031 OR RO5073031	64
#35	tirzepatide OR LY3298176 OR LY-3298176	281
#36	#22 OR #23 OR #24 OR #25 OR #26 OR #27 OR #28 OR #29 OR #30 OR #31 OR #32 OR #33 OR #34 OR #35	26858
#37	#21 AND #36	17801
#38	Filters applied: Clinical Study, Clinical Trial, Clinical Trial, Phase II, Clinical Trial, Phase III, Clinical Trial, Phase IV, Comparative Study, Controlled Clinical Trial, Evaluation Study, Pragmatic Clinical Trial, Randomized Controlled Trial, Humans, Adult: 19+ years, Young Adult: 19-24 years, Adult: 19-44 years, Middle Aged + Aged: 45+ years, Middle Aged: 45-64 years, Aged: 65+ years, 80 and over: 80+ years.	2363

S2 Table Systematic Review Search Strategy (Continued)

**Cochrane Library (From Inception to September 02, 2023)**		
**Search**	**Query**	**Items Found**
#1	MeSH descriptor: [Obesity] explode all trees	21415
#2	MeSH descriptor: [Overweight] explode all trees	24827
#3	MeSH descriptor: [Body Weight Changes] explode all trees	11248
#4	MeSH descriptor: [Weight Loss] explode all trees	8188
#5	MeSH descriptor: [Body Weight] explode all trees	41393
#6	MeSH descriptor: [Body Composition] explode all trees	7136
#7	MeSH descriptor: [Body Fat Distribution] explode all trees	1209
#8	MeSH descriptor: [Body Mass Index] explode all trees	12436
#9	MeSH descriptor: [Waist-Height Ratio] explode all trees	16
#10	MeSH descriptor: [Waist-Hip Ratio] explode all trees	317
#11	(“body mass index” OR BMI OR “body weight”):ti,ab,kw AND (over):ti,ab,kw	29451
#12	(weight):ti,ab,kw AND (loss OR lost OR losing OR reduc* OR gain* OR increase* OR program*):ti,ab,kw	94432
#13	(obes* OR overweight OR “over-weight” OR “weight loss” OR “weight reduction” OR “body mass index” OR “high BMI” OR “BMI above”):ti,ab,kw	98841
#14	#1 OR #2 OR #3 OR #4 OR #5 OR #6 OR #7 OR #8 OR #9 OR #10 OR #11 OR #12 OR #13	162179
#15	MeSH descriptor: [Diabetes Mellitus, Type 2] explode all trees	23347
#16	MeSH descriptor: [Diabetic Nephropathies] explode all trees	1800
#17	(“diabetes mellitus type 2” OR “diabetes mellitus” OR NIDDM):ti,ab,kw	79773
#18	(“diabetic nephropath*” OR “diabetic kidney disease”):ti,ab,kw	621
#19	(diabetes OR “diabetes mellitus” OR diabetic*):ti,ab,kw AND (“type 2” OR “type II” OR “type ii” OR “non-insulin dependent” OR “noninsulin dependent” OR “adult onset” OR “mature onset” OR “late onset”):ti,ab,kw	51446
#20	#15 OR #16 OR #17 OR #18 OR #19	87107
#21	#14 OR #20	223221
#22	MeSH descriptor: [Glucagon-Like Peptide 1] explode all trees	2196
#23	MeSH descriptor: [Gastric Inhibitory Polypeptide] explode all trees	439
#24	(“glucagon receptor” OR “glucagon like peptide 1” OR “glucose-dependent insulinotropic polypeptide”):ti,ab,kw	4306
#25	(“glucagon-like peptide” OR “glucagon-like peptide 1” OR “GLP-1” OR GLP1):ti,ab,kw AND (receptor* OR stimulat* OR inhibitor* OR agonist* OR “long act*”):ti,ab,kw	147
#26	(“glucose-dependent insulinotropic polypeptide” OR GIP):ti,ab,kw AND (inhibitor* OR agonist*):ti,ab,kw	686
#27	albiglutide OR GSK-716155 OR GSK716155	155
#28	dulaglutide OR LY-2189265 OR LY2189265	555
#29	efpeglenatide OR HM-11260C OR SAR-439977 OR SAR439977	40
#30	exenatide OR “exendin 4” OR AC-2993 OR AC2993 OR LY-2148568 OR LY2148568	1404
#31	liraglutide OR NN-2211 OR NN2211	2324
#32	lixisenatide OR AVE-010 OR AVE-0010 OR AVE0010 OR AVE010	385
#33	semaglutide OR NN-9535 OR NN9535 OR NN9924 OR NN-9924	1022

#34	taspoglutide OR BIM51077 OR RO-5073031 OR RO5073031	55
#35	tirzepatide OR LY3298176 OR LY-3298176	188
#36	#22 OR #23 OR #24 OR #25 OR #26 OR #27 OR #28 OR #29 OR #30 OR #31 OR #32 OR #33 OR #34 OR #35	8107
#37	#21 AND #36	7053
#38	MeSH descriptor: [Child] explode all trees	78477
#39	MeSH descriptor: [Adolescent] explode all trees	125806
#40	MeSH descriptor: [Infant] explode all trees	41997
#41	#38 OR #39 OR #40	190517
#42	#37 NOT #41	6755
#43	MeSH descriptor: [Randomized Controlled Trials as Topic] explode all trees	47401
#44	MeSH descriptor: [Random Allocation] explode all trees	23366
#45	MeSH descriptor: [Clinical Trial] explode all trees	45348
#46	MeSH descriptor: [Controlled Clinical Trial] explode all trees	38478
#47	(“randomi$ed controlled trial*” OR “controlled clinical trial*” OR “random allocation” OR “double-blind method” OR “single-blind method” OR “clinical trial”):ti,ab,kw	465365
#48	(control* OR clinical OR allocated):ti,ab,kw AND (trial* OR random* OR RCT* OR placebo*):ti,ab,kw	1489467
#49	(singl* OR doubl* OR trebl* OR tribl*):ti,ab,kw AND (blind* OR mask* dumm*):ti,ab,kw	404548
#50	#43 OR #44 OR #45 OR #46 OR #47 OR #48 OR #49	1509858
#51	#42 AND #50	5888
#52	#51 in Trials	5866

S2 Table Systematic Review Search Strategy (Continued)

**Scopus (From Inception to September 02, 2023)**		
**Search**	**Query**	**Items Found**
#1	TITLE-ABS-KEY (obes* OR overweight OR “over-weight” OR “body weight change*” OR “weight loss” OR “weight reduction” OR “body weight” OR “body composition*” OR “body fat distribution” OR “body mass index” OR “high BMI” OR “BMI above” OR “waist-height ratio” OR “waist-hip ratio”)	1531199
#2	TITLE-ABS-KEY ((“body mass index” OR BMI OR “body weight”) AND over)	136043
#3	TITLE-ABS-KEY (weight AND (loss OR lost OR losing OR reduc* OR gain* OR increase* OR program*))	1589617
#4	#1 OR #2 OR #3	2426639
#5	TITLE-ABS-KEY (“diabetes mellitus type 2” OR “diabetes mellitus” OR “diabetic Nephropath*” OR NIDDM OR “diabetic nephropath*” OR “diabetic kidney disease”)	959576
#6	TITLE-ABS-KEY ((diabetes OR “diabetes mellitus” OR diabetic*) AND (“type 2” OR “type II” OR “type ii” OR “non-insulin dependent” OR “noninsulin dependent” OR “adult onset” OR “mature onset” OR “late onset”))	352122
#7	#5 OR #6	991403
#8	#4 OR #7	3155820
#9	TITLE-ABS-KEY (“glucagon receptor” OR “glucagon like peptide 1” OR “glucagon-like peptide 1” OR “glucose-dependent insulinotropic polypeptide” OR “gastric inhibitory polypeptide”)	35610
#10	TITLE-ABS-KEY ((“glucagon-like peptide” OR “glucagon-like peptide 1” OR “GLP-1” OR GLP1) AND (receptor* OR stimulat* OR inhibitor* OR agonist* OR “long act*”))	28367
#11	TITLE-ABS-KEY ((“glucose-dependent insulinotropic polypeptide” OR GIP) AND (inhibitor* OR agonist*))	4170
#12	TITLE-ABS-KEY (albiglutide OR “GSK-716155” OR GSK716155)	1239
#13	TITLE-ABS-KEY (dulaglutide OR “LY-2189265” OR LY2189265)	2186
#14	TITLE-ABS-KEY (efpeglenatide OR “HM-11260C” OR “SAR-439977” OR SAR439977)	129
#15	TITLE-ABS-KEY (exenatide OR “exendin 4” OR “AC-2993” OR AC2993 OR “LY-2148568” OR LY2148568)	10141
#16	TITLE-ABS-KEY (liraglutide OR “NN-2211” OR NN2211)	9999
#17	TITLE-ABS-KEY (lixisenatide OR “AVE-010” OR “AVE-0010” OR AVE0010 OR AVE010)	1971
#18	TITLE-ABS-KEY (semaglutide OR “NN-9535” OR NN9535 OR NN9924 OR “NN-9924”)	2997
#19	TITLE-ABS-KEY (taspoglutide OR BIM51077 OR “RO-5073031” OR RO5073031)	240
#20	TITLE-ABS-KEY (tirzepatide OR LY3298176 OR “LY-3298176”)	542
#21	#9 OR #10 OR #11 OR #12 OR #13 OR #14 OR #15 OR #16 OR #17 OR #18 OR #19 OR #20	45772
#22	#8 AND #21	33862
#23	TITLE-ABS-KEY (child* OR adolescent OR infant OR p?ediatric)	5645606
#24	#22 AND NOT #23	32150

#25	TITLE-ABS-KEY (“randomi?ed controlled trial*” OR “random allocation” OR “clinical trial*” OR “controlled clinical trial*” OR “double-blind method” OR “single-blind method”)	2298219
#26	TITLE-ABS-KEY ((control* OR clinical OR allocated) AND (trial* OR random* OR RCT* OR placebo*))	3296474
#27	TITLE-ABS-KEY ((singl* OR doubl* OR trebl* OR tribl*) AND (blind* OR mask* dumm*))	2989
#28	#25 OR #26 OR #27	3327851
#29	#24 AND #28	11616
#30	#29 AND (LIMIT-TO (DOCTYPE , "ar")) AND (LIMIT-TO (SRCTYPE , "j"))	6446

S2 Table Systematic Review Search Strategy (Continued)

**CINAHL (From Inception to September 02, 2023)**		
**Search**	**Query**	**Items Found**
#1	MH obesity or overweight or fat or obese or unhealthy weight or high bmi	94065
#2	MH body weight changes	1251
#3	MH weight loss	24895
#4	MH body weight	35276
#5	MH body composition or body fat or fat free mass or lean body mass	21785
#6	MH body mass index	95738
#7	MH waist circumference or waist measurement	7448
#8	AB ("body mass index" OR BMI OR "body weight") AND AB over	17797
#9	AB weight AND AB (loss OR lost OR losing OR reduc* OR gain* OR increase* OR program*)	114430
#10	AB obes* OR overweight OR "over-weight" OR "weight loss" OR "weight reduction" OR "body mass index" OR "high BMI" OR "BMI above"	172247
#11	S1 OR S2 OR S3 OR S4 OR S5 OR S6 OR S7 OR S8 OR S9 OR S10	337933
#12	MH diabetes mellitus	
#13	AB "diabetes mellitus type 2" OR "diabetes mellitus" OR NIDDM OR "diabetic nephropath*" OR "diabetic kidney disease"	49957
#14	AB (diabetes OR "diabetes mellitus" OR diabetic*) AND AB ("type 2" OR "type II" OR "type ii" OR "non-insulin dependent" OR "noninsulin dependent" OR "adult onset" OR "mature onset" OR "late onset")	52869
#15	S12 OR S13 OR S14	143931
#16	S11 OR S15	445991
#17	MH glucagon-like peptide-1 receptor agonists	874
#18	AB "glucagon receptor" OR "glucagon like peptide 1" OR "glucose-dependent insulinotropic polypeptide"	1368
#19	AB ("glucagon-like peptide" OR "glucagon-like peptide 1" OR "GLP-1" OR GLP1) AND AB (receptor* OR stimulat* OR inhibitor* OR agonist* OR "long act*")	2118
#20	AB ("glucose-dependent insulinotropic polypeptide" OR GIP) AND AB (inhibitor* OR agonist*)	240
#21	TX albiglutide OR "GSK-716155" OR GSK716155	233
#22	TX dulaglutide OR "LY-2189265" OR LY2189265	542
#23	TX efpeglenatide OR "HM-11260C" OR "SAR-439977" OR SAR439977	29
#24	TX exenatide OR "exendin 4" OR "AC-2993" OR AC2993 OR "LY-2148568" OR LY2148568	2143
#25	TX liraglutide OR "NN-2211" OR NN2211	2216
#26	TX lixisenatide OR "AVE-010" OR "AVE-0010" OR AVE0010 OR AVE010	455
#27	TX semaglutide OR "NN-9535" OR NN9535 OR NN9924 OR "NN-9924"	746
#28	TX taspoglutide OR BIM51077 OR "RO-5073031" OR RO5073031	51
#29	TX tirzepatide OR LY3298176 OR "LY-3298176"	166
#30	S17 OR S18 OR S19 OR S20 OR S21 OR S22 OR S23 OR S24 OR S25 OR S26 OR S27 OR S28 OR S29	6935
#31	S16 AND S30	4114
#32	MH children or adolescents or youth or child or teenager	526266
#33	MH infant	190917

#34	S32 OR S33	620996
#35	S31 NOT S34	4040
#36	MH randomized controlled trials or rtc or randomised control trials	138956
#37	AB “randomi?ed controlled trial*” OR “controlled clinical trial*” OR “random allocation” OR “double-blind method” OR “single-blind method” OR “clinical trial”	139586
#38	AB (control* OR clinical OR allocated) AND AB (trial* OR random* OR RCT* OR placebo*)	390224
#39	AB (singl* OR doubl* OR trebl* OR tribl*) AND AB (blind* OR mask* dumm*)	53565
#40	S36 OR S37 OR S38 OR S39	451810
#41	S35 AND S40	1341
#42	Limiters - Randomized Controlled Trials<br>Narrow by Subject Age: aged, 80 & over, adult: 19-44 years, aged: 65+ years, middle aged: 45-64 years, all adult<br>Expanders - Apply equivalent subjects	406
