# Supplementary material for: Comparative effectiveness of glucagon-like peptide-1 receptor agonists on body composition and anthropometric indices: A protocol for a systematic review and network meta-analysis of randomized controlled trials
Source: PLoS One. 2024 Feb 26;19(2):e0297488. doi: 10.1371/journal.pone.0297488 (PMC10896528; doi:10.1371/journal.pone.0297488)
Supplement: S3 Table — (PDF) [file pone.0297488.s003.pdf]

S3 Table Grey Literature From Ongoing Clinical Trial Register

**Ongoing clinical trial register**
• Australia and New Zealand's (ANZCTR) (http://www.anzctr.org.au)<br>• Brazilian Clinical Trials Registry (ReBec) (http://www.ensaiosclinicos.gov.br)<br>• Chinese Clinical Trial Registry (ChiCTR) (http://www.chictr.org.cn)<br>• Clinical Research Information Service (CRiS), Republic of Korea (http://cris.cdc.go.kr)<br>• Clinical Trials Registry - India (CTRI) (http://ctri.nic.in)<br>• Cuban Public Registry of Clinical Trials(RPCEC) (http://registroclinico.sld.cu)<br>• EU Clinical Trials Register (EU-CTR) (https://www.clinicaltrialsregister.eu)<br>• German Clinical Trials Register (DRKS) (http://www.drks.de)<br>• Iranian Registry of Clinical Trials (IRCT) (http://www.irct.ir)<br>• Japan Primary Registries Network (https://rctportal.niph.go.jp)<br>• The Netherlands Trial Register (http://www.trialregister.nl)<br>• Pan African Clinical Trial Registry (PACTR) (http://www.pactr.org)<br>• Peruvian Registry of Clinical Trials (http://www.ins.gob.pe/ensayosclinicos)<br>• Philippine Health Research Registry (http://registry.healthresearch.ph)<br>• Sri Lanka Clinical Trials Registry (SLCTR) (http://www.slctr.lk)<br>• South African National Clinical Trials Register (http://www.sanctr.gov.za)<br>• Swiss FOPH Human Research Projects (https://www.kofam.ch/en/swiss-clinical-trials-portal.html)<br>• Tanzania Clinical Trial Registry (http://www.tzctr.or.tz)<br>• Thai Clinical Trials Registry (http://www.clinicaltrials.in.th)<br>• The United Kingdoms' ISRCTN registry (http://www.isrctn.com)<br>• The US National Institutes of Health Ongoing Trials Registry (http://clinicaltrials.gov)<br>• The World Health Organization International Clinical Trials Registry Platform (ICTRP) (https://www.who.int/ictrp)
**Preprint databases**
• medRxiv (https://www.medrxiv.org)<br>• bioRxiv (https://www.biorxiv.org)<br>• Research Square (https://www.researchsquare.com)
